# Does (re-)entering the labour market at advanced ages protect against cognitive decline? A matching difference-in-differences approach

**DOI:** 10.1136/jech-2022-220197

**Published:** 2023-07-17

**Authors:** Jung Hyun Kim, Graciela Muniz-Terrera, Anja K Leist

**Affiliations:** 1 Department of Social Sciences, University of Luxembourg, Institute for Research on Socio-Economic Inequality, Esch-sur-Alzette, Luxembourg; 2 Department of Social Medicine, Ohio University, Athens, Ohio, USA; 3 Centre for Clinical Brain Sciences, The University of Edinburgh, Edinburgh, UK

**Keywords:** aging, cognition, employment, public health, social sciences

## Abstract

**Background:**

While prolonged labour market participation becomes increasingly important in ageing societies, evidence on the impacts of entering or exiting work beyond age 65 on cognitive functioning is scarce.

**Methods:**

We use data from two large population-representative data sets from South Korea and the USA to investigate and compare the effects of the labour market (re-)entry and exit by matching employment and other confounder trajectories prior to the exposure. We chose the Korean Longitudinal Study of Aging (N=1872, 2006–2020) for its exceptionally active labour participation in later life and the Health and Retirement Study (N=4070, 2006–2020) for its growing inequality among US older adults in labour participation. We use the matching difference-in-differences (DID) method, which allows us to make causal claims by reducing biases through matching.

**Results:**

We find general positive effects of entering the labour market in South Korea (DID estimate: 0.653, 95% CI 0.167 to 1.133), while in the USA such benefit is not salient (DID estimate: 0.049, 95% CI −0.262 to 0.431). Exiting the late-life labour market leads to cognitive decline in both South Korea (DID estimate: −0.438, 95% CI −0.770 to –0.088) and the USA (DID estimate: −0.432, 95% CI −0.698 to –0.165).

**Conclusions:**

Findings suggest that Korean participants cognitively benefited from late-life labour market participation, while US participants did not. Differences in participant characteristics and reasons for labour market participation may have led to the differential findings. We found the negative effects of exiting the late-life labour force in both countries.

**WHAT IS ALREADY KNOWN ON THIS TOPIC**

- Previous research has established the negative relationship between retirement and cognitive function. Labour force participation and exit beyond normative retirement age (65+) and the relationship with cognitive function is less studied.

**WHAT THIS STUDY ADDS**

- We provide evidence from large population-representative South Korean data, with active late-life labour market participation and compare results with the USA. Entering the labour market at age 65+ positively affects cognitive function in South Korea but not in the USA. We find negative effects of exiting the labour market at age 65+ in both South Korea and the USA.

**HOW THIS STUDY MIGHT AFFECT RESEARCH, PRACTICE OR POLICY**

- This research indicates measures to facilitate labour market participation at advanced ages in South Korea may help delay cognitive decline. Future research is needed to investigate whether such positive benefit continues to exist with increasing levels of educational attainment and living conditions.

## Introduction

An increase in advanced-age labour force participation, specifically beyond ages 65 and older, has been observed across industrialised economies over the last decade.^1–4^ South Korea makes itself an interesting case study with the highest labour force participation rate of older adults, accounting for 36% of the individuals aged 65+ in 2021.^5^

In this paper, we examine the impacts of the late-life labour market ‘entry and exit’ on cognitive function in South Korea and compare the causal effects with results from the USA, a country whose older population has marked cultural and socioeconomic characteristics with South Korea’s. We provide contextual comparisons of both countries on late-adulthood financial conditions in online supplemental appendix 1.

10.1136/jech-2022-220197.supp1 Supplementary data

Our main interest is cognitive function. Poor cognitive function is a growing public health concern for ageing societies.^6^ A decline in cognitive function is negatively associated with the deprivation of one’s physical and mental autonomy and imposes a financial burden on the family and society due to the high cost of health and social care.^7^ Due to such reasons, exiting or remaining in the labour force at later adulthood on cognitive function has received public health and economic attention.^8–14^ Yet, very few studies have examined the impacts of late-life labour market participation beyond age 65, and the evidence has been restricted to a few countries.

This paper uses population-based data from South Korea and the USA. Investigating and comparing causal effects with more than one population is challenging with age-non-related policy change as an instrument unless such changes occur in parallel. To overcome these limitations, we use the matching difference-in-differences from the potential outcomes framework.^15^ This approach shares the reasoning of trial emulation in epidemiology, which mimics randomised controlled trials.^16 17^ This method matches the confounders and the employment histories prior to the exposure. Past work history is an important confounder affecting future employment status^18^ and cognitive health.^19^ By matching according to the employment history, we may capture further unobserved time-varying confounders such as work attitude, desire to work or job insecurity.

In line with the ‘use it or lose it’ hypothesis,^20^ which intellectually stimulating activities can protect against cognitive decline in later life, we expect labour market entry to have positive effects on cognitive functioning and labour market exits to have negative effects, similar across countries. We explored putative moderators, sex/gender, education and socioeconomic status without directed hypotheses.

## Methods

### Study population

**Figure 1:**
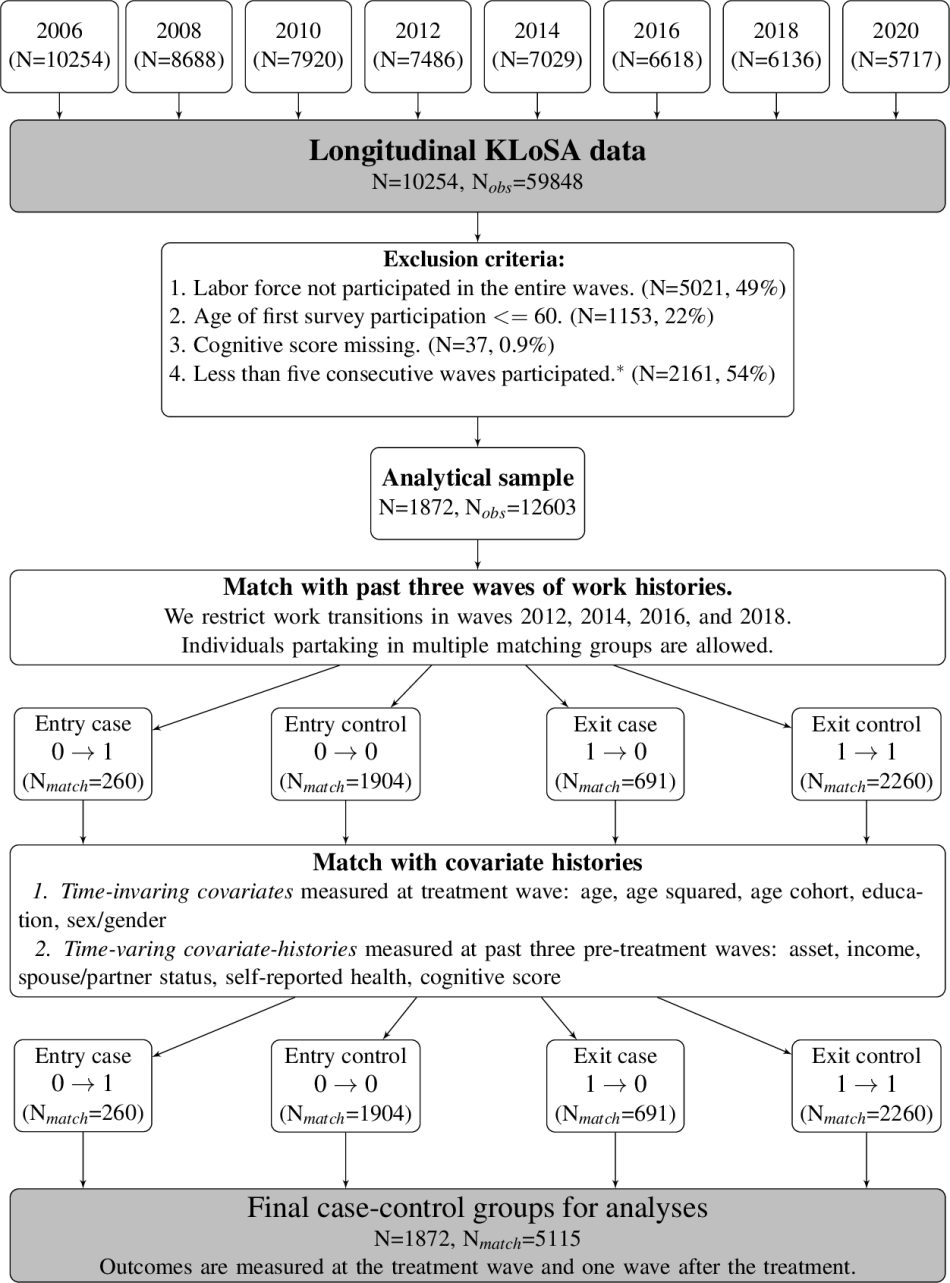
Flowchart of a final analytical KLoSA sample This flowchart summarises matching steps and how we arrive at our final sample size. N is the number of individuals, N_obs_ is the number of observations (period-person), and N match is the number of matched observations. ⇤We require the fourth criterion to match up to three past waves of employment histories and examine up to one wave following the transition. Missing patterns of this criterion are provided in the online supplemental table 4. KLoSA, Korean Longitudinal Study of Aging.

Data came from the Korean Longitudinal Study of Aging (KLoSA), which is a sister study of the Health Retirement Study (HRS). It started data collection in 2006 and it is designed to be nationally representative of Korean households. It is a biennial survey on approximately 10 000 individuals on demographics, family composition, health, employment and financial status for adults over age 45 who reside in South Korea (excluding Jeju Island). Further information is found on the KLoSA website (http://survey.keis.or.kr). We used data from 2006 to 2020 for this study (figure 1).

**Figure 2:**
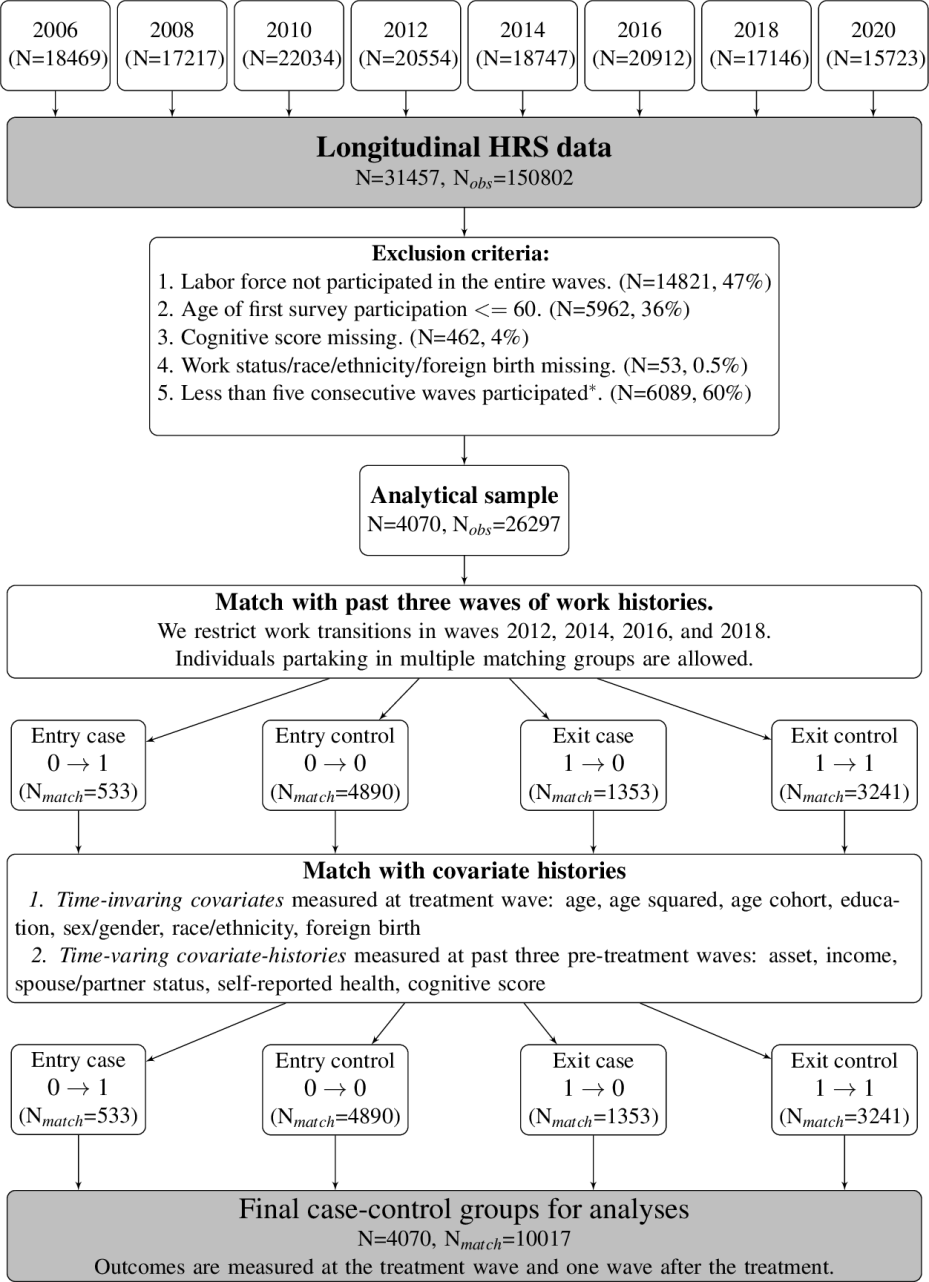
Flowchart of a final analytical HRS sample This flowchart summarises matching steps and how we arrive at our final sample size. N is the number of individuals, N_obs_ is the number of observations (period-person), and N match is the number of matched observations. ⇤We require the fifth criterion to match up to three past waves of employment histories and examine up to one wave following the transition. Missing patterns of this criterion are provided in the online supplemental table 5. HRS, Health Retirement Study.

For the US analysis, we used the HRS, a nationally representative sample of private households with members aged 51 years and older in the US since 1992. It is a biennial follow-up data on more than 43 000 individuals on demographics, family structure, health and economic resources. Further information is available elsewhere.^21^ We used from year 2006 to 2018 of the RAND HRS Longitudinal File 2018 (V.2) and the HRS 2020 Core Early Release (V.2.0) (figure 2).

### Outcome measures

In KLoSA, the Korean version of the Mini-Mental State Examination (K-MMSE) is used to measure global cognitive function. It takes integer values between 0 and 30, with higher values indicating better functioning. K-MMSE is a modified version of MMSE adjusted to the older Korean population.^22^

In HRS, the Telephone Interview for Cognitive Status (TICS) is applied to measure global cognitive function.^23^ TICS is modelled after MMSE for large-scale population-based cognitive assessment via telephone or face-to-face administration.^24^ It takes integer values between 0 and 27, with higher values indicating better functioning.

Comparisons of the composition of these two measurements and the distributions of each measurement are found in online supplemental table 1 and figure 1. Several studies argued that TICS and MMSE scores correlate very highly.^24 25^ The estimated effect cannot be interpreted in terms of magnitude, yet it is sufficient to present the direction of the effect.

### Employment status transitions at late life

To reduce possible selection into entering or exiting the labour market, we employ a difference-in-differences (DID) design to compare participants with and without exposure after several steps of reducing possible confounding. Using the terminology of the DID method, the so-called ‘treatment’ ‘entering the labour market’ captures employment transitions from being non-employed at wave *t−*1 to employed at wave *t*. We compare individuals entering the labour market to the ‘control’ group of individuals who remain inactive, that is, out of the labour market from wave *t−*1 to wave *t*. Likewise, ‘exiting the labour market’ identifies employment transitions from being employed at wave *t−*1 to non-employed at wave *t*. We compare individuals who exit the labour market to the ‘control’ group of individuals who stay in the labour market from wave *t−*1 to wave *t*. We restrict the treatment years from 2012 to 2018.

### Covariates

Following the established definitions, we call all methods that balance the covariates between the treated and control groups ‘matching’.^26^ We match the following variables: age, age squared, sex/gender, education, household net income, household net asset, occupation level, living with a spouse/partner, self-reported health, a birth year before 1945 and cognitive scores. For HRS, we additionally match race/ethnicity and foreign birth for its availability in the data. Time-invariant variables are measured at the treatment year, and the time-varying variables are measured throughout the last three waves prior to the transitions to capture the trajectories. We classified occupations based on the skill levels following the International Standard Classification of Occupations.^27^ Financial values such as asset and income are harmonised into US$ in thousands adjusted for purchasing power parity^28^ and inflation.^29^ In the analyses, we transformed asset and income values to tertiles as proxies for relative economic status. Covariates with missing values are handled by creating indicator variables of partially observed variables with values 0 for the missing values and 1 for the non-missing values. Table 1 describes the baseline characteristics of each of the analytical samples used. Online supplemental tables 2 and 3 show the descriptive statistics by entry to or exit from the labour market for both data sets.

**Table 1:** Descriptive statistics of HRS and KLoSA

	HRS N=4070	KLoSA N=1872	*P* value	N
Cognitive function (HRS: TICS, KLoSA: MMSE)	16.6 (3.88)	25.9 (4.06)	0	5942
Age	65.5 (4.94)	65.0 (4.58)	*<*0.001	5942
Age category:			0.015	5942
−64	2203 (54.1%)	1083 (57.9%)		
65–69	1078 (26.5%)	487 (26.0%)		
70–74	536 (13.2%)	213 (11.4%)		
75–79	237 (5.82%)	86 (4.59%)		
85-	16 (0.39%)	3 (0.16%)		
Birth year≤1945	2596 (63.8%)	1151 (61.5%)	0.094	5942
Female	2194 (53.9%)	816 (43.6%)	*<*0.001	5942
Education:			0	5932
Up to primary	122 (3.00%)	1038 (55.4%)		
Secondary	209 (5.15%)	306 (16.3%)		
High school	1565 (38.5%)	392 (20.9%)		
Above high school	2164 (53.3%)	136 (7.26%)		
Spouse/partner	2885 (70.9%)	1572 (84.0%)	*<*0.001	5941
Household asset	547.3 (1205.8)	163.0 (263.0)	*<*0.001	4860
Annual income	78.4 (143.6)	14.0 (15.1)	*<*0.001	5888
Occupation level:			*<*0.001	4023
Elementary	325 (11.2%)	303 (27.2%)		
Service/skilled-manual	1629 (56.0%)	728 (65.4%)		
Managerial/professional	956 (32.9%)	82 (7.37%)		
Self-reported health:			*<*0.001	5940
Very bad	67 (1.65%)	385 (20.6%)		
Bad	535 (13.2%)	595 (31.8%)		
Fair	1351 (33.2%)	724 (38.7%)		
Good	1495 (36.8%)	139 (7.43%)		
Very good	620 (15.2%)	29 (1.55%)		
Race/ethnicity:			.	4070
Non-Hispanic white	2964 (72.8%)	. (.)		
Non-Hispanic black	642 (15.8%)	. (.)		
Hispanic	362 (8.89%)	. (.)		
Non-Hispanic others	102 (2.51%)	. (.)		
Foreign birth	417 (10.2%)	. (.)	.	4070

All covariates are measured at the study entry regardless of waves. Listed values are mean (*±* SD) or total number (%).

Cognitive functions are measured by HRS: HRS-TICS, KLoSA: K-MMSE.

HRS sample uses education year to calculate each category; −6, 7–9, 10–12, >12 years. Asset/income is harmonised into thousands USD with inflation/PPP adjustment. Occupation level is calculated with last observation due to high missingness.

Race/ethnicity and foreign birth are not asked in the KLoSA survey.

Source: HRS 2006-2020, KLoSA 2006-2020 own calculations.

HRS, Health Retirement Study; KLoSA, Korean Longitudinal Study of Aging; K-MMSE, Korean version of the Mini-Mental State Examination; TICS, Telephone Interview for Cognitive Status.

### Statistical analysis

We apply the matching DID method.^15^ It first matches ‘control’ observations with identical employment histories in the same period as the ‘treatment group’. Borrowing from Imai, Kim and Wang^15^, we refer to the set of matched ‘control’ observations as a *matched set*.^15^ Online supplemental figure 2 presents a graphical illustration of matching across individuals and waves. Then, it refines the matched sets via weighting using pretreatment covariate histories up to three past waves prior to the transitions. Finally, it computes the DID estimators among refined matched sets.

We chose the number of lags to be three to include more than one wave of pre-‘treatment’ history while balancing against the need for a sufficient sample size of the matched set. We set the number of leads to be one to have enough individuals in the matched set and to avoid effects from interference coming from the lead period. We present a separate sensitivity analysis with a lag of two waves pre-‘treatment’ in online supplemental appendix 2.

### Covariate balancing using pre-treatment covariate trajectories

We first match individuals by their employment histories and build a matched set. Subsequently, we balance the covariates of the ‘control’ and the ‘treatment’ group. This is done by giving higher weights to individuals in the matched set with similarity in terms of covariate history to the treatment group. We tested several covariate balancing methods, such as Mahalanobis distance matching,^30^ propensity score matching,^31^ propensity score weighting and covariate balancing propensity score (CBPS),^32^ and CBPS weighting method best adjusted the covariates (online supplemental appendix 3).

### Assumptions

Following the covariate balancing, three assumptions need to be satisfied. The most challenging one is the *parallel trend assumption*, which needs to be met to ensure that the effect is driven by the treatment and not by possible unobserved confounding in the pretreated period. Visual inspections (online supplemental figures 3 and 4) indicate that the parallel assumption might be valid, as the standardised mean difference in cognitive score of the pre-exposed period after balancing was close to zero.

The second assumption is the absence of *spillover effects*, which means that one’s employment status transition should not affect others’ cognitive function. We cannot rule out the possibility of spillover effects as we do not have information on the connectedness of individuals through living in close geographical proximity or sharing the work environment, etc. However, we believe that the amount is trivial.

Finally, although this method allows the investigation of *carry-over effects* by deciding the number of lags to consider, we must assume that the potential outcome is independent of the treatment history beyond the number of lags, three waves. We believe that employment histories of up to three waves (6 years) are enough to capture unobserved confounders related to employment status.

### Model estimation

We present the empirical DID estimation according to Imai *et al*.^15^ Briefly, $DID_{Entry}\left(F,3\right)$ is the average causal effect measured at wave F after entering the labour market, assuming that the cognitive function depends on the work history up to three waves back. This study focuses on the causal quantity measured in the treatment wave and one wave after, $DID\left(0,3\right)$ and $DID\left(1,3\right)$ .

Specifically, in (1), i is a case observation, $i^{`}$ is a ‘control’ observation and t is time. $M_{it}$ is the number of observations in the matched set. $w_{it}^{i^{`}}$ is the non-negative weight constructed from matched set constituting the ‘control’ group with CBPS weighting. $Entry_{it}$ is an indicator function that has value 1 if the individual entered the labour market and has any positive number of individuals from the matched set. *N* is the number of observations.

(1) $$\begin{array}{ll} {\hat{DID}}_{Entry}\left(F,3\right)= & \frac{1}{\sum_{i=1}^{N}\sum_{t=4}^{T-F}Entry_{it}}Entry_{it}\\ & \left[\left(Cog_{i,t+F}-Cog_{i,t-1}\right)-\sum_{i^{\prime }\in M_{it}}w_{it}^{i^{\prime }}\left(Cog_{i^{\prime },t+F}-Cog_{i^{\prime },t-1}\right)\right] \end{array}$$

where $Cog_{i,t+F}-Cog_{i,t-1}$ is the difference in cognitive score between time t-1 and t+F for the case observations that entered the labour market. Whereas $w_{it}^{i^{`}}\left(Cog_{i^{`},t+F}-Cog_{i^{`},t-1}\right)$ is the counterfactual, the weighted difference in cognitive scores for the ‘control’ observations who are out of labour market but sharing identical past employment history with the case observations. Likewise, we build separate matched sets for the exit case, ${\hat{DID}}_{Exit}$ where $E{xit}_{it}$ becomes the exposure.

SEs of the estimator from (1) are calculated with 1000 repetitions of the weighted block bootstrap procedures.^15 33^ The method described above was implemented using an open-source statistical software package *PanelMatch* 34 in R V.4.2.1 (R Foundation for Statistical Computing, Vienna, Austria). STATA V.17.0 (StataCorp LLC, College Station, Texas) was used for data preparation.

## Results

### Sample characteristics

Our final samples include 1872 Korean individuals and 4070 US individuals (figures 1 and 2). We present descriptive statistics comparing Korea and USA in table 1. US participants reported higher assets and incomes than Korean participants. A noticeable difference between the two samples was that the share of education above the high school level was more than seven times higher in the US sample. While only 7% of the Korean sample had managerial or professional occupations, 33% of US participants belonged to this category of occupation group. Descriptive statistics according to the transition status are provided in online supplemental tables 2 and 3.

### Estimated effects of entering and exiting the labour market

**Figure 3:**
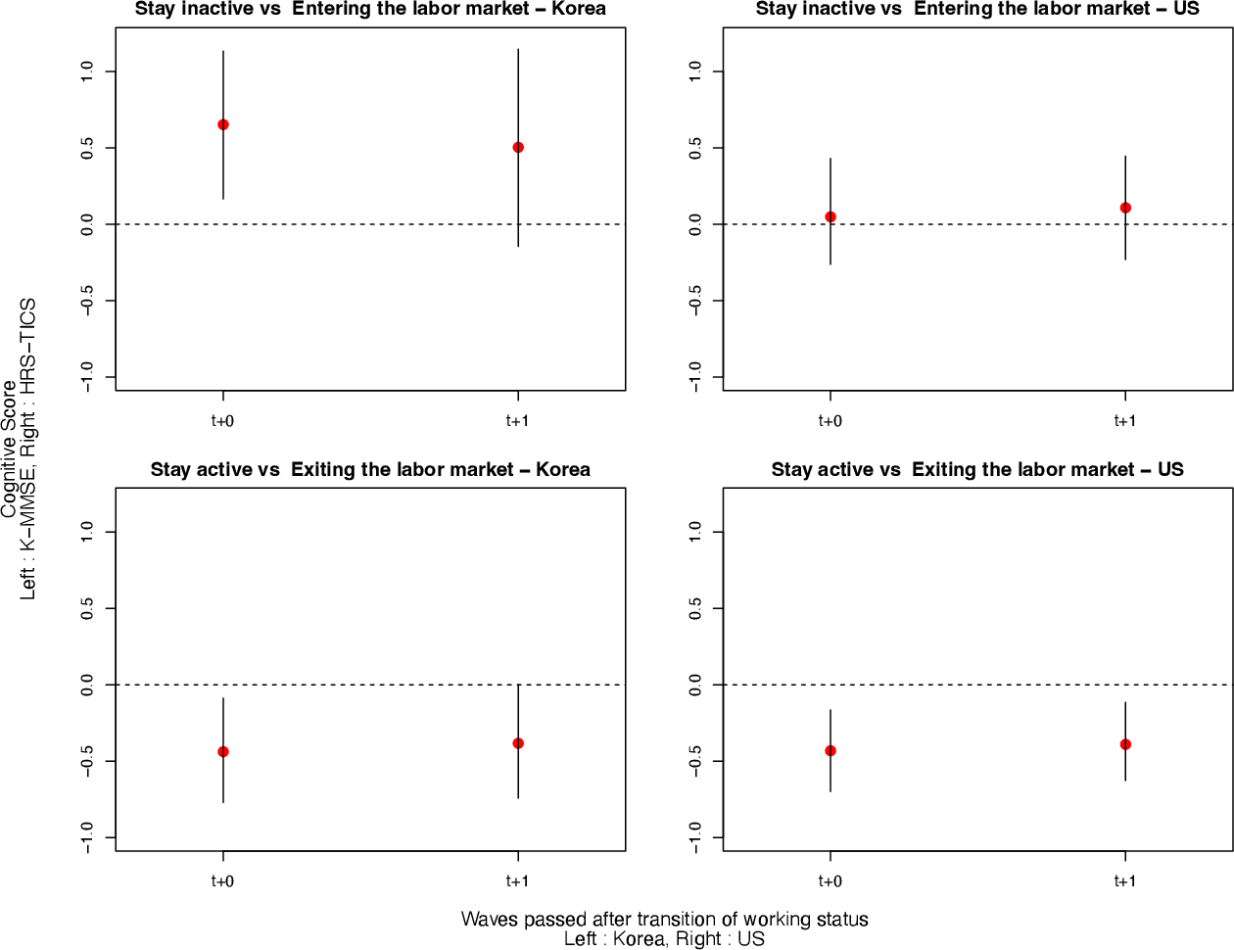
Estimated effects of entry to and exit from the late-life labour market on cognitive function in KLoSA and the HRS sample The estimation results are obtained after matching according to treatment history and covariate balancing propensity score (CBPS) weighting with covariate histories during the three waves before the treatment. The left panel indicates the results from the Korean sample and the right panel is from the US sample. The estimates for the average effects of entering the labour market (upper panel) and exiting (bottom panel) are shown for the period of immediate and one wave after the transition, with 95% asymptotic CI as vertical bars. CBPS weighting is chosen for its best performance in adjustment. HRS, Health Retirement Study; KLoSA, Korean Longitudinal Study of Aging; K-MMSE, Korean version of the Mini-Mental State Examination; TICS, Telephone Interview for Cognitive Status.

Figure 3 shows the estimated effects of entering the labour market and exiting on cognitive functioning for immediate and the wave following the transition in the Korean and the US sample. The effects of entering the labour market were positive during the transition wave in the Korean sample, but such effects were not found in the US sample. Meanwhile, we found negative effects in both samples.

Table 2 compares the unadjusted and adjusted estimation results. The magnitude of the positive effects of entering the labour market in Korea was reduced but remained positive after covariate balancing. We observe a negative effect of exiting in both samples before and after the covariate balancing. We show that our main results are robust to the matching with shorter lags in online supplemental appendix 2.

**Table 2:** Estimated effects of late-life labour transitions on cognitive function

	Cognitive function							
	Unadjusted				Adjusted			
	DID	SE	2.50%	97.50%	DID	SE	2.50%	97.50%
Entering the labour market								
Korea at t+0	0.920*	0.242	0.462	1.39	0.653*	0.251	0.167	1.133
Korea at t+1	0.828*	0.315	0.224	1.484	0.504	0.331	−0.144	1.146
US at t+0	0.213	0.178	−0.143	0.551	0.049	0.18	−0.262	0.431
US at t+1	0.318	0.176	−0.021	0.671	0.108	0.171	−0.23	0.446
Exiting the labour market								
Korea at t+0	−0.456*	0.197	−0.857	−0.07	−0.438*	0.173	−0.77	−0.088
Korea at t+1	−0.418	0.209	−0.833	0.033	−0.383	0.19	−0.741	0.002
US at t+0	−0.473*	0.129	−0.718	−0.208	−0.432*	0.137	−0.698	−0.165
US at t+1	−0.466*	0.126	−0.717	−0.218	−0.390*	0.132	−0.627	−0.116

DID: difference-in-differences; estimation of the average effects of labour transition on cognition.

Adjustment: Covariates are measured prior to the transitions; age, age squared, sex/gender, education, household net income, household net asset, occupation level, living with a spouse/partner, self-reported health, a birth year before 1945 and past cognitive scores. In the analysis of US data, we include race/ethnicity and foreign birth.

SE, Weighted bootstrapped standard errors with 1000 repetitions. 2.5%, 97.5%, 95% asymptotic CI, *p<0.05.

We used covariate balancing propensity score (CBPS) weighting for adjustment.

Source: KLoSA 2006-2020, HRS 2006-2020, own calculations.

### Subgroup analyses by socioeconomic status and sex/gender

We show the DID estimates based on subgroup analyses by median asset level measured at the study entry wave, education level, and sex/gender (online supplemental figures 5–7). Due to its relatively large sample size, we conduct subgroup analyses solely with HRS data. We found individuals with below-median baseline asset level, low education and men experienced more noticeable negative effects from exiting the labour market. We did not observe such differences for the entry into the late-life labour market.

## Discussion

An increase in advanced-age labour force participation in ageing countries calls for the need to understand better the impacts of labour market participation and withdrawal at ages beyond 65 years on cognitive functioning. The present study examines labour market ‘entry and exit’ effects on cognitive functioning using data from two large population representative studies, HRS and its sister Korean study KLoSA.

Using the matching difference-in-differences that follows the idea of trial emulation,^15–17^ we find that the effects of employment transitions at age 65+ on cognitive function are heterogeneous in country contexts. The estimated effects of entering the labour market were positive in Korea, while we did not find such positive effects in the USA. On the other hand, we found negative effects from exiting the labour market in both data sets. To understand the magnitude of the positive effects from the Korean study, we compare our result to a study that investigated the effects of receiving social pension for 5 years on cognitive functioning using the same data and cognitive assessment.^35^ They found a positive effect of 1.309 points while in our study estimated a positive effect of 0.653 points for entering the late-life labour market, roughly half the size of the cognitive benefit from receiving long-term social pension.

Our results add support to the ‘use it or lose it’ hypothesis in late-life labour force participation^20^ and are in line with previous studies of the positive associations of labour market participation at advanced ages and cognitive functioning.^12–14^ However, our findings suggest that the general positive effects are country specific. We discuss two potential mechanisms that might explain the unique positive effects of entry into the labour market in Korea in online supplemental appendix 4. Concerning the well-established detrimental effects of labour market withdrawal,^8–10^ our study comes to the same conclusion as previous studies by using a different causal identification strategy. We add to this body of literature by extending the study population from retirees to anyone exiting the labour market exit at 65+, potentially including postretirement work. Furthermore, we show that negative effects are more pronounced in groups with low socioeconomic status and in men.

There are limitations to this study. First, the sample size of the Korean study is relatively small and, thus, does not allow for subgroup analyses. Furthermore, the ‘treated’ group in our analyses represents a rather small portion of individuals who transitioned into or out of work in Korea and the USA. Concerning the possibility of reverse causality, we present online supplemental tables 2 and 3, which measured cognitive function one wave before the transitions. We observe that in both countries, individuals who enter the labour force are not positively selected in terms of better cognitive functioning. Rather they have the second lowest cognitive functioning, contrary to the reverse causality argument. Second, we remove a large share of observations (USA: 60%; Korea: 54%) due to the criterion of five consecutive participation in the survey. This exclusion is crucial to match individuals with past three employment histories and investigate the effects up to one follow-up wave. We report the descriptive statistics by the exclusion criterion in online supplemental tables 4 and 5. A sensitivity analysis relaxing this criterion from five to four waves of consecutive participation by matching on past two waves led to similar result patterns (online supplemental appendix 2). Third, the two cognitive measurements in each data set are overlapping in some dimensions but are not identical. Compared with the US data, the distribution in Korean data is more skewed to the right. While we believe some of the concerns are relieved by using the *change* score of cognitive functioning instead of the score itself, we suggest refraining from making direct comparisons in the magnitude of the effects until further harmonisation of the cognitive assessments becomes available. Regarding data collection changes due to the COVID-19 outbreak, in 2020, HRS opted to use telephone testing exclusively instead of randomising individuals for in-person assessments for cognition. While there could be mode effects, HRS had applied random assignment to in-person versus telephone testing in precedent waves and showed no differences in performance. KLoSA administered cognitive testing in person for all waves, including 2020, with similar response rates. Fourth, our analytical strategy is subjected to unobserved time-varying confounders that might influence the labour force transitions and cognitive functioning such as somatic disorders which are not captured by conventional health measures. Fifth, both cognitive scores measure global cognition and are known to have poor sensitivity to small changes in cognitive functioning.

Despite these limitations, our contribution to the knowledge of employment status transitions at advanced ages and cognitive functioning is analysing the transition effects with a rigorous modelling strategy and a cross-country perspective from South Korea, where one out of three is participating in the labour market at 65+,^5^ and the USA, with growing inequality among older adults in labour participation.^36^ Estimating identical data-analytic models with multiple data sets is useful for the external validity of findings by ensuring replicability and reproducibility of the research design.^37 38^ This is promising with a growing attempt to harmonise better cognitive ageing data from different countries.^39^

Future research should add by testing more potential pathways and confounders of effects of labour market entry and exit by assessing possible effect differences in more fine-grained types of occupation and psychosocial work characteristics, health conditions, and racial/ethnic groups.

## Data Availability

Data are available in a public, open access repository. The Korean Longitudinal Study of Ageing (KLoSA) data can be accessed by the public upon member registration at https://survey.keis.or.kr/eng/klosa/klosa01.jsp. However, please note that the KLoSA has temporarily ceased public access to the Mini-Mental Examination Score (MMSE) data for the 2020 wave due to the need for internal review for data disclosure (https://survey.keis.or.kr/madang/notice/Read.jsp?ntt_id=5634, announced in April 2023). The Health and Retirement Study (HRS) data are publicly available upon member registration at https://hrs.isr.umich.edu/data-products. The code used in this paper is accessible in Zenodo digital repository at https://zenodo.org/record/8023732.
