# Supplementary material for: Does (re-)entering the labour market at advanced ages protect against cognitive decline? A matching difference-in-differences approach
Source: J Epidemiol Community Health. 2023 Jul 17;77(10):663–9. doi: 10.1136/jech-2022-220197 (PMC10511963; doi:10.1136/jech-2022-220197)
Supplement: Supplementary data [file jech-2022-220197supp001.pdf]

Supplementary Material

Does (re-)entering the labor market at advanced ages protect against cognitive decline? A matching difference-in-differences approach

Contents

1 Supplementary appendix 3

1. Late-life financial conditions in South Korea and the US 3
2. Sensitivity analysis with shorter lags 4
3. Different matching methods 6
4. Potential mechanisms 7

2 Tables 8

Table 1. Cognitive measurement comparison 8

Table 2. Descriptive statistics by labor force transition in KLoSA 9

Table 3. Descriptive statistics by labor force transition in HRS 11

Table 4. Descriptive statistics by survey participation in KLoSA at entry of the study 13

Table 5. Descriptive statistics by survey participation in HRS at the entry of the study 15

3 Figures 17

Figure 1. Distribution of cognitive score 17

Figure 2. Graphical illustration of employment history matching 18

Figure 3. Covariate balance in KLoSA 19

Figure 4. Covariate balance in HRS 20

Figure 5. Subgroup analyses by baseline median asset level with HRS sample. 21

Figure 6. Subgroup analyses by education level with HRS sample. 22

Figure 7. Subgroup analyses by sex/gender level with HRS sample. 23

1. Late-life financial conditions in South Korea and the US

According to the latest report [1], earnings from work as a source of income account for more than half of the total income of older adults aged 65+ in Korea. This is the second-largest share of work income contributing to total income among all OECD countries, exceeded only by Mexico. The share of public transfers on total income at ages 65+ is only slightly above 25% in Korea, while public transfers contribute to 57% of advanced-age incomes in the average OECD countries (In Korea, the pension age was 60 in 2007 and 62 in 2020 [1, 2]). Moreover, a low percentage of private occupation-related pensions as a source of income excludes the possibility of private pensions substituting the lack of public transfers. This suggests that the maturing of the public pension system has not yet fully managed to keep pace with the country's earning growth. Among OECD countries, Korea ranked highest in the share of older adults in relative income poverty, defined by having an income below half the national median equalized household disposable income [1]. With few alternative income sources to compensate for insufficient public transfers, a large share of Korean older adults (re-)enters the labor market at advanced ages. However, the generational income gap still remains between the current working-age population and the population aged 65+.

In the US, earnings from work account for around 35% of income sources of older adults, which is roughly 10% higher than the OECD average. More than 40% of the total income is covered by public transfers (In the US, the normal retirement age was between 65 and 66 in 2004. It is 66 years and eight months for workers aged 62 in 2020 [1, 2]. Retirees on average have around 94% of the average total income of the total population, which is higher than the OECD average. The labor force participation of older adults in the US aged 65+ was 18.9% in 2021, 3.4% higher than the average OECD countries [3]. Overall, older adults in the US are better financially than the average older adults across OECD countries. However, an alarming amount of income inequality measured by the Gini coefficient implies that the favorable conditions of older adults are disproportionately shared [1].

Kim JH, *et al. J Epidemiol Community Health* 2023; 77:663–669. doi: 10.1136/jech-2022-220197

2. Sensitivity analysis with shorter lags

A robustness check with a shorter length of employment and covariate history prior to the entry to or exit from the labor market is performed in Figure 1. Shorter employment and covariate history windows will allow more individuals to be included in the matched set, which leads to reduced variation in the estimation. However, we need to assume that the potential cognitive function only depends on the past two waves of working history and that it is enough to capture unobserved confounders related to employment status. Therefore, balancing with shorter waves leaves room for potential confounders from past histories beyond two waves. We redid the main analyses with two waves of pre-treatment histories as sensitivity analysis acknowledging this trade-off.

The estimated coefficients move in the same direction once we match with shorter waves of lags. The positive effect of entering the labor market in Korea holds and is even larger and lasting with shorter lags adjustment. The negative effects of exit hold for both samples. All analyses are in the same direction as the main analysis.

Kim JH, *et al. J Epidemiol Community Health* 2023; 77:663–669. doi: 10.1136/jech-2022-220197

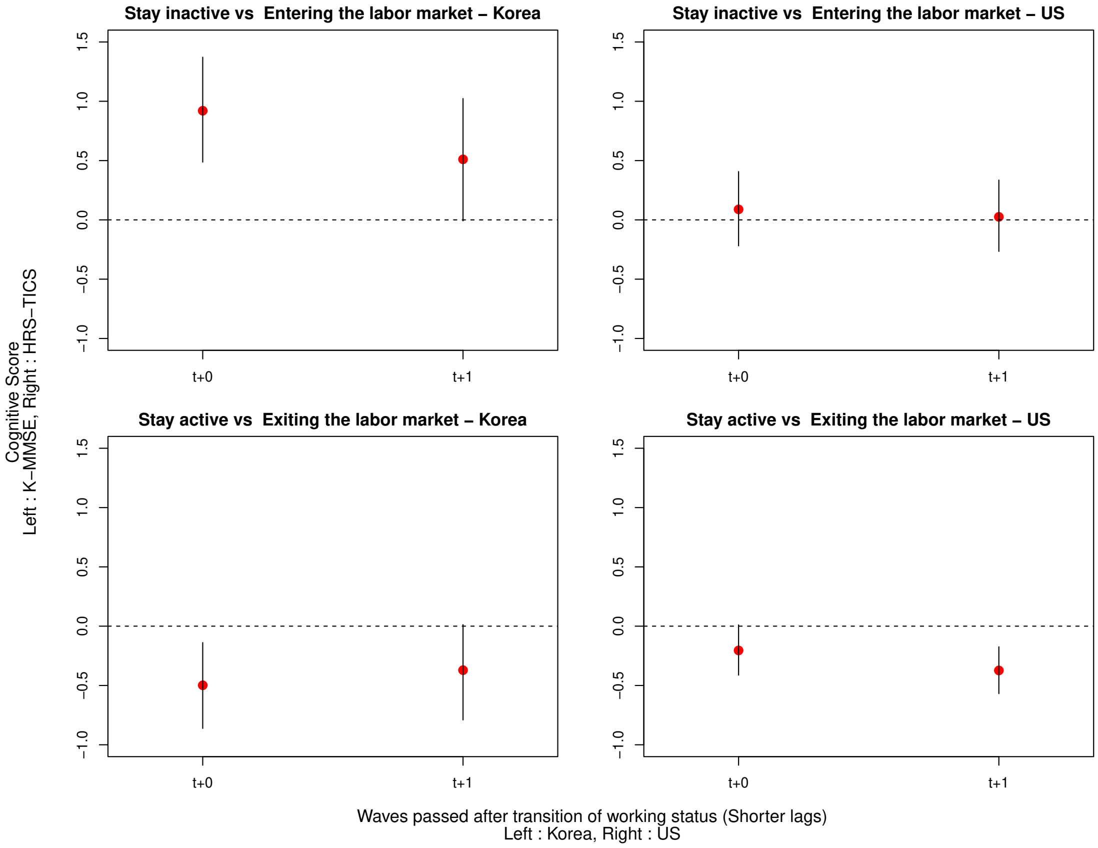

Figure 1: **Estimated effects of entry to and exit from the late-life labor market on cognitive function with shorter lags** The estimation results are obtained after matching according to treatment history and covariate balancing propensity score (CBPS) weighting with covariate histories during the two waves before the treatment. The left panel indicates the results from the Korean sample and the right panel from the US sample. The estimates for the average effects of entering the labor market (upper panel) and exiting (bottom panel) are shown for the period of immediate and one wave after the transition, with 95% asymptotic confidence intervals as vertical bars. CBPS weighting is chosen for its best performance in adjustment.

Kim JH, *et al. J Epidemiol Community Health* 2023; 77:663–669. doi: 10.1136/jech-2022-220197

3. Different matching methods

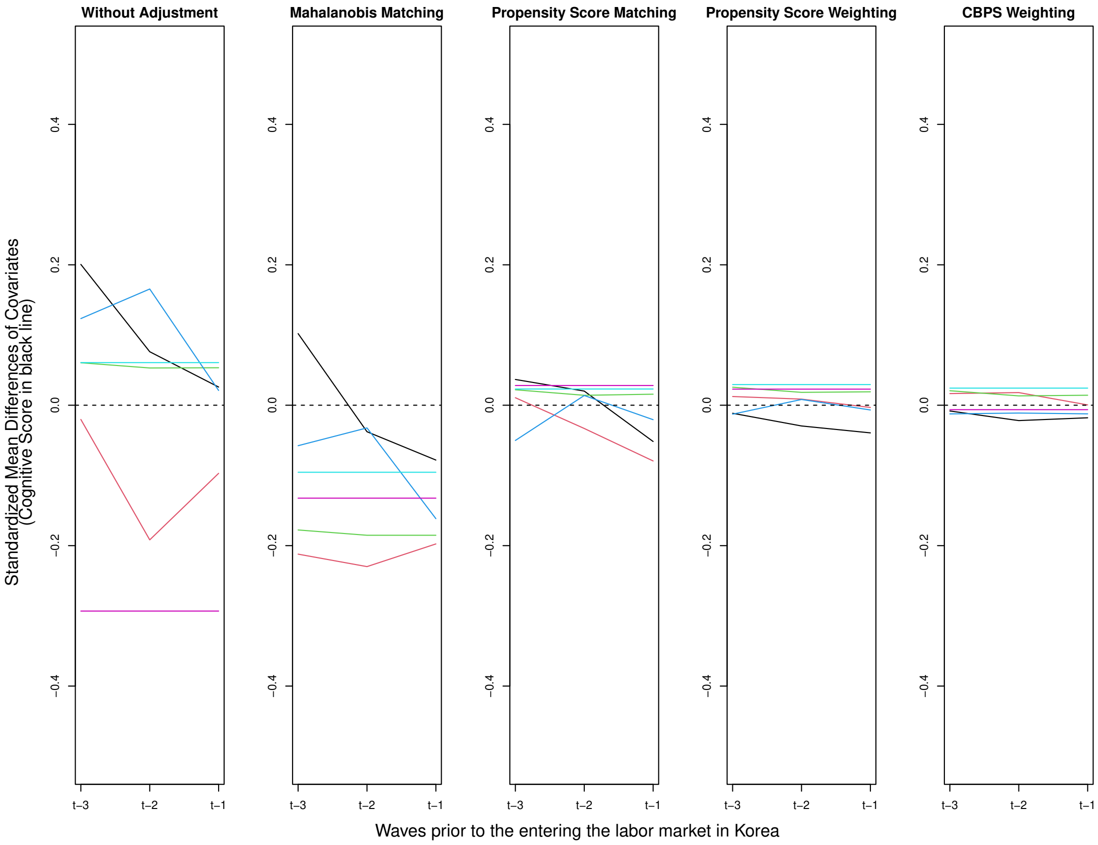

Figure 2: **Covariate balances comparisons in KLoSA of entering the labor market** Each plot presents the standardized mean difference of covariates over the pre-treatment time period with KLoSA data for the case of entering the labor market. The first column represents the unadjusted balance, and the next four columns compare the different balancing methods. The black line represents the balance of the lagged cognitive scores, whereas the colored lines represent highlighted covariates; age (purple), health (red), education (green), asset (blue), and female (light blue).

4. Potential mechanisms

In the Korean sample, we found positive effects of entering the labor market on cognitive function. These general effects observed uniquely in the Korean sample might be due to several reasons.

First, KLoSA participants have lower education, income, and asset level compared to the HRS respondents. Moreover, it is individuals with lower cognitive scores and socioeconomic status within the country enter the late-life labor market. In Korea, people with lower education and occupational class were more likely to work after retirement [4, 5]. The cognitive gain might be related to the absence of cognitively stimulating activities outside of the workplace due to insufficient financial means that create a relative cognitive benefit at the workplace.

Secondly, the positive effects of entering the labor market in the Korean sample might be due to general psychological benefits from working in this cultural context. Studies from geographically and culturally close countries provide relevant evidence. One study from Singapore showed that participants who continued working after retirement had fewer depressive symptoms than those exiting the labor market permanently [6]. Another study found that Japanese men who started working at post-retirement ages had fewer depressive symptoms [7]. It is known that retirees who experienced severe work identity loss were more likely to intend to reenter the labor force [8, 9]. Self-identity culturally strongly tied to work might explain the roots of the benefit beyond monetary ones.

Table 1: Cognitive measurement

Data	KLoSA	HRS
Measurement (point)	K-MMSE (30)	HRS-TICS (27)
Immediate word recall (3|10)	✓(three words)	✓(ten words)
Delayed word recall (3|10)	✓(three words)	✓(ten words)
Serial 7s (5)	✓	✓
Backwards counting (2)		✓
Date (5|4)	✓	
Place (5)	✓	
Language (9)	✓	

Table 2: Descriptive statistics by labor force transition in KLoSA

	Entry case N_{obs}=260	Entry control N_{obs}=1904	Exit case N_{obs}=691	Exit control N_{obs}=2260	*P value*	N_{obs}
K-MMSE	24.8 (4.60)	24.6 (4.87)	25.0 (4.55)	25.8 (4.07)	<0.001	5115
Age	71.0 (4.65)	72.5 (5.33)	72.2 (5.38)	70.8 (4.57)	<0.001	5115
Age Category:					.	5115
65-69	122 (46.9%)	647 (34.0%)	255 (36.9%)	1042 (46.1%)		
70-74	71 (27.3%)	640 (33.6%)	228 (33.0%)	740 (32.7%)		
75-79	57 (21.9%)	405 (21.3%)	139 (20.1%)	376 (16.6%)		
80-84	9 (3.46%)	166 (8.72%)	54 (7.81%)	86 (3.81%)		
85-	1 (0.38%)	46 (2.42%)	15 (2.17%)	16 (0.71%)		
Birth Year<1945	176 (67.7%)	1450 (76.2%)	531 (76.8%)	1609 (71.2%)	<0.001	5115
Female	140 (53.8%)	930 (48.8%)	319 (46.2%)	886 (39.2%)	<0.001	5115
Education:					0.042	5115
Up to Primary	157 (60.4%)	1083 (56.9%)	428 (61.9%)	1328 (58.8%)		
Secondary	43 (16.5%)	288 (15.1%)	102 (14.8%)	371 (16.4%)		
High School	42 (16.2%)	380 (20.0%)	119 (17.2%)	436 (19.3%)		
Above High School	18 (6.92%)	153 (8.04%)	42 (6.08%)	125 (5.53%)		
Spouse/Partner	201 (77.3%)	1391 (73.1%)	516 (74.7%)	1914 (84.7%)	<0.001	5115
Household Asset:					0.001	5115
Low	98 (37.7%)	682 (35.8%)	262 (37.9%)	753 (33.3%)		
Middle	103 (39.6%)	600 (31.5%)	242 (35.0%)	783 (34.6%)		
High	59 (22.7%)	622 (32.7%)	187 (27.1%)	724 (32.0%)		
Household Income:					<0.001	5098
Low	108 (41.7%)	776 (40.9%)	211 (30.6%)	552 (24.5%)		
Middle	79 (30.5%)	589 (31.1%)	270 (39.1%)	902 (40.0%)		
High	72 (27.8%)	531 (28.0%)	209 (30.3%)	799 (35.5%)		
Occupation Level:					<0.001	4088
Elementary	60 (48.8%)	565 (40.5%)	243 (40.6%)	565 (28.7%)		
Service/Skilled-Manual	58 (47.2%)	730 (52.3%)	325 (54.3%)	1291 (65.5%)		
Managerial/Professional	5 (4.07%)	101 (7.23%)	31 (5.18%)	114 (5.79%)		
Self-Reported Health:					.	5115
Poor	66 (25.4%)	568 (29.8%)	161 (23.3%)	414 (18.3%)		

continued on next page

Table 2 – *continued from previous page*

	Entry case N_{obs}=260	Entry control N_{obs}=1904	Exit case N_{obs}=691	Exit control N_{obs}=2260	*P value*	N_{obs}
Fair	104 (40.0%)	778 (40.9%)	297 (43.0%)	959 (42.4%)		
Good	78 (30.0%)	500 (26.3%)	215 (31.1%)	782 (34.6%)		
Very good	10 (3.85%)	53 (2.78%)	15 (2.17%)	96 (4.25%)		
Excellent	2 (0.77%)	5 (0.26%)	3 (0.43%)	9 (0.40%)		

Notes: All covariates are measured at one wave prior to the transition.

Listed values are mean (± standard deviation) or total number (%).

Asset/income are inflation adjusted.

Occupation level is calculated with last observation due to high missingness.

Source: KLoSA 2006-2020, own calculations.

Kim JH, *et al. J Epidemiol Community Health* 2023; 77:663–669. doi: 10.1136/jech-2022-220197

Table 3: Descriptive statistics by labor force transition in HRS

	Entry case N_{obs}=533	Entry control N_{obs}=4890	Exit case N_{obs}=1353	Exit control N_{obs}=3241	*P value*	N_{obs}
HRS-TICS	15.5 (3.93)	15.3 (3.99)	15.8 (3.96)	16.2 (3.90)	<0.001	10017
Age	71.4 (5.22)	72.6 (5.56)	71.7 (5.18)	71.2 (4.95)	<0.001	10017
Age category:					<0.001	10017
-64	10 (1.88%)	42 (0.86%)	19 (1.40%)	57 (1.76%)		
65-69	216 (40.5%)	1667 (34.1%)	501 (37.0%)	1341 (41.4%)		
70-74	189 (35.5%)	1540 (31.5%)	469 (34.7%)	1079 (33.3%)		
75-79	80 (15.0%)	1034 (21.1%)	250 (18.5%)	547 (16.9%)		
80-84	25 (4.69%)	478 (9.78%)	86 (6.36%)	181 (5.58%)		
85-	13 (2.44%)	129 (2.64%)	28 (2.07%)	36 (1.11%)		
Birth Year<1945	397 (74.5%)	3694 (75.5%)	1002 (74.1%)	2362 (72.9%)	0.061	10017
Female	264 (49.5%)	2815 (57.6%)	725 (53.6%)	1632 (50.4%)	<0.001	10017
Education:					<0.001	10003
up to 6 yrs	13 (2.44%)	163 (3.34%)	30 (2.22%)	77 (2.38%)		
7-9 yrs	28 (5.25%)	247 (5.06%)	82 (6.07%)	143 (4.42%)		
10-12 yrs	212 (39.8%)	2139 (43.8%)	543 (40.2%)	1108 (34.3%)		
> 12 yrs	280 (52.5%)	2337 (47.8%)	696 (51.5%)	1905 (58.9%)		
Spouse/Partner	348 (65.5%)	3071 (62.8%)	891 (65.9%)	2186 (67.5%)	<0.001	10010
Household Asset:					<0.001	10017
Low	197 (37.0%)	1707 (34.9%)	474 (35.0%)	995 (30.7%)		
Middle	166 (31.1%)	1727 (35.3%)	463 (34.2%)	1077 (33.2%)		
High	170 (31.9%)	1456 (29.8%)	416 (30.7%)	1169 (36.1%)		
Household Income:					<0.001	10017
Low	215 (40.3%)	2130 (43.6%)	388 (28.7%)	701 (21.6%)		
Middle	176 (33.0%)	1703 (34.8%)	486 (35.9%)	1115 (34.4%)		
High	142 (26.6%)	1057 (21.6%)	479 (35.4%)	1425 (44.0%)		
Occupation Level:					<0.001	8311
Elementary	57 (17.1%)	474 (12.0%)	134 (11.8%)	290 (9.98%)		
Service/Skilled-Manual	178 (53.3%)	2343 (59.5%)	645 (56.9%)	1689 (58.1%)		
Managerial/Professional	99 (29.6%)	1120 (28.4%)	354 (31.2%)	928 (31.9%)		
Self-Reported Health:					<0.001	10010

continued on next page

Kim JH, *et al. J Epidemiol Community Health* 2023; 77:663–669. doi: 10.1136/jech-2022-220197

Table 3 – *continued from previous page*

	Entry case N_{obs}=533	Entry control N_{obs}=4890	Exit case N_{obs}=1353	Exit control N_{obs}=3241	*P value*	N_{obs}
Very Bad	10 (1.88%)	190 (3.89%)	24 (1.78%)	26 (0.80%)		
Bad	76 (14.3%)	882 (18.1%)	194 (14.3%)	356 (11.0%)		
Fair	193 (36.2%)	1830 (37.5%)	501 (37.1%)	1045 (32.3%)		
Good	191 (35.8%)	1613 (33.0%)	509 (37.6%)	1372 (42.4%)		
Very Good	63 (11.8%)	371 (7.59%)	124 (9.17%)	440 (13.6%)		
Race/Ethnicity:					0.020	10017
Non-Hispanic white	405 (76.0%)	3537 (72.3%)	1018 (75.2%)	2457 (75.8%)		
Non-Hispanic black	68 (12.8%)	792 (16.2%)	206 (15.2%)	455 (14.0%)		
Hispanic	49 (9.19%)	437 (8.94%)	101 (7.46%)	245 (7.56%)		
Non-Hispanic other	11 (2.06%)	124 (2.54%)	28 (2.07%)	84 (2.59%)		
Foreign birh:	47 (8.82%)	525 (10.7%)	133 (9.83%)	290 (8.95%)	0.050	10017

Notes: All covariates are measured at one wave prior to the transition.

Listed values are mean (± standard deviation) or total number (%).

Asset/income are inflation adjusted.

Occupation level is calculated with last observation due to high missingness.

Source: HRS 2006-2020, own calculations.

Table 4: Descriptive statistics by survey participation >= 5 in KLoSA at the entry of the study

	< 5 waves N=2161	>= 5 waves N=1872	*P value*	N
K-MMSE	27.0 (3.58)	25.9 (4.01)	<0.001	4033
Age	63.0 (3.71)	64.9 (4.46)	<0.001	4033
Age category:			.	4033
-64	1830 (84.7%)	1115 (59.6%)		
65-69	175 (8.10%)	476 (25.4%)		
70-74	102 (4.72%)	202 (10.8%)		
75-79	35 (1.62%)	59 (3.15%)		
80-84	12 (0.56%)	18 (0.96%)		
85-	7 (0.32%)	2 (0.11%)		
Birth Year<1945:	444 (20.5%)	1151 (61.5%)	<0.001	4033
Female:	941 (43.5%)	816 (43.6%)	1.000	4033
Education:			<0.001	4033
Up to Primary	656 (30.4%)	1038 (55.4%)		
Secondary	431 (19.9%)	306 (16.3%)		
High School	800 (37.0%)	392 (20.9%)		
Above High School	274 (12.7%)	136 (7.26%)		
Spouse/Partner:	1863 (86.2%)	1578 (84.3%)	0.095	4033
Household Asset:			<0.001	2450
Low	448 (26.1%)	255 (34.8%)		
Middle	525 (30.6%)	248 (33.9%)		
High	745 (43.4%)	229 (31.3%)		
Household Income:			<0.001	3950
Low	373 (17.5%)	698 (38.5%)		
Middle	622 (29.1%)	573 (31.6%)		
High	1141 (53.4%)	543 (29.9%)		
Occupation Level:			0.001	2460
Elementary	431 (32.1%)	302 (27.1%)		
Service/Skilled-Manual	780 (58.0%)	731 (65.5%)		
Managerial/Professional	133 (9.90%)	83 (7.44%)		
Self-Reported Health:			<0.001	4033
Poor	264 (12.2%)	386 (20.6%)		

continued on next page

Kim JH, *et al. J Epidemiol Community Health* 2023; 77:663–669. doi: 10.1136/jech-2022-220197

Table 4 – *continued from previous page*

	Less than 5 waves N=2161	>= 5 waves N=1872	*P value*	N
Fair	746 (34.5%)	597 (31.9%)		
Good	966 (44.7%)	720 (38.5%)		
Very good	166 (7.68%)	140 (7.48%)		
Excellent	19 (0.88%)	29 (1.55%)		

Notes: All covariates are measured at the study entry regardless of waves.

Listed values are mean (± standard deviation) or total number (%).

Less than 5 waves is a group that participated survey less than five waves.

Asset/income are inflation adjusted.

Occupation level is calculated with last observation due to high missingness.

Sources: KLoSA 2006-2020, own calculations.

Table 5: Descriptive statistics by survey participation >= 5 in HRS at the entry of the study

	< 5 waves N=6089	>= 5 waves N=4070	*P value*	N
HRS-TICS	16.1 (4.23)	16.6 (3.87)	<0.001	10159
Age	63.3 (4.28)	65.5 (4.90)	<0.001	10159
Age category:			<0.001	10159
-64	5079 (83.4%)	2217 (54.5%)		
65-69	495 (8.13%)	1075 (26.4%)		
70-74	259 (4.25%)	530 (13.0%)		
75-79	146 (2.40%)	196 (4.82%)		
80-84	77 (1.26%)	36 (0.88%)		
85-	33 (0.54%)	16 (0.39%)		
Birth Year<1945:	1036 (17.0%)	2596 (63.8%)	0.000	10159
Female:	3150 (51.7%)	2194 (53.9%)	0.033	10159
Education:			<0.001	10103
up to 6 yrs	299 (4.95%)	122 (3.00%)		
7-9 yrs	254 (4.20%)	209 (5.15%)		
10-12 yrs	2066 (34.2%)	1565 (38.5%)		
> 12 yrs	3424 (56.7%)	2164 (53.3%)		
Spouse/Partner:	3563 (69.0%)	2892 (71.1%)	0.032	9236
Household Asset:			<0.001	9238
Low	2029 (39.3%)	1182 (29.0%)		
Middle	1676 (32.4%)	1360 (33.4%)		
High	1463 (28.3%)	1528 (37.5%)		
Household Income:			<0.001	9238
Low	1573 (30.4%)	972 (23.9%)		
Middle	1555 (30.1%)	1415 (34.8%)		
High	2040 (39.5%)	1683 (41.4%)		
Occupation Level::			0.001	5282
Elementary	302 (12.8%)	328 (11.2%)		
Service/Skilled-Manual	1196 (50.7%)	1636 (56.0%)		
Managerial/Professional	860 (36.5%)	960 (32.8%)		
Self-Reported Health::			<0.001	9232
Very Bad	196 (3.80%)	67 (1.65%)		

continued on next page

Table 5 – *continued from previous page*

	Less than 5 waves N=6089	>= 5 waves N=4070	*P value*	N
Bad	995 (19.3%)	537 (13.2%)		
Fair	1709 (33.1%)	1340 (32.9%)		
Good	1707 (33.1%)	1499 (36.8%)		
Very Good	557 (10.8%)	625 (15.4%)		
Race/Ethnicity:			<0.001	10159
Non-Hispanic white	3633 (59.7%)	2964 (72.8%)		
Non-Hispanic black	1261 (20.7%)	642 (15.8%)		
Hispanic	972 (16.0%)	362 (8.89%)		
Non-Hispanic other	223 (3.66%)	102 (2.51%)		
Foreign birh:	1003 (16.5%)	417 (10.2%)	<0.001	10159

Notes: All covariates are measured at the study entry regardless of waves.

Listed values are mean (± standard deviation) or total number (%).

Less than 5 waves is a group that participated survey less than five waves.

Asset/income are inflation adjusted.

Occupation level is calculated with last observation due to high missingness.

Sources: HRS 2006-2020, own calculations.

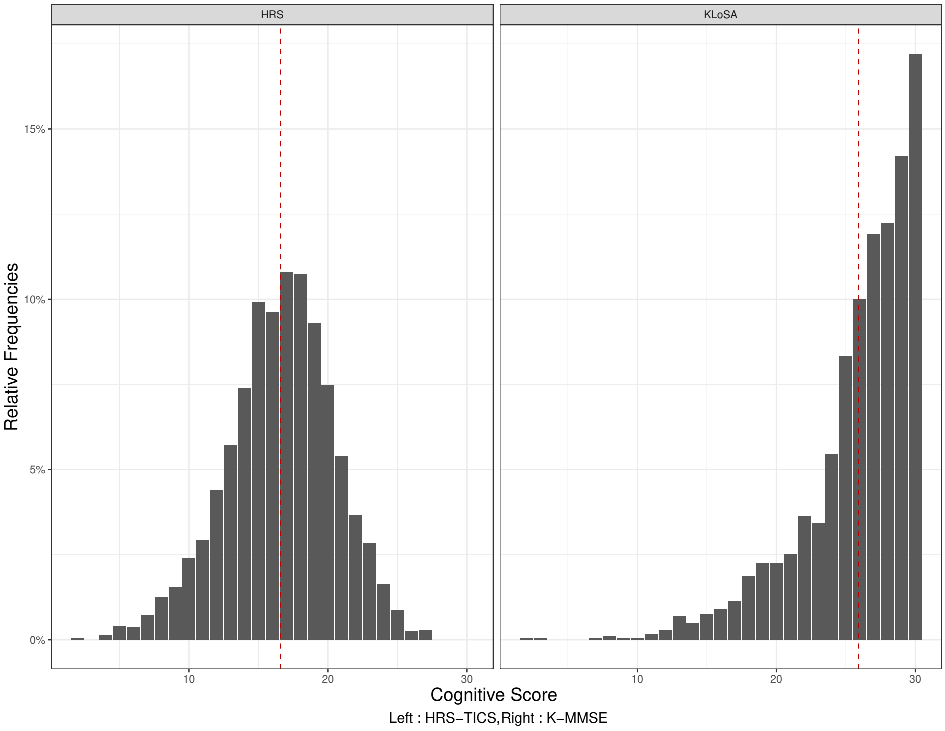

Figure 1: **Distribution of cognitive score** The bar graphs show the distribution of relative frequencies in each value of cognitive score within each data set. The left panel indicates the distribution of US data. Cognitive function is measured by HRS-TICS ranging from 0 to 27. The right panel displays the distribution of Korean data. Cognitive function is measured by K-MMSE, ranging from 0 to 30. For both measurements, higher values indicate better function. The red dotted line represents the average cognitive score.

Kim JH, *et al. J Epidemiol Community Health* 2023; 77:663–669. doi: 10.1136/jech-2022-220197

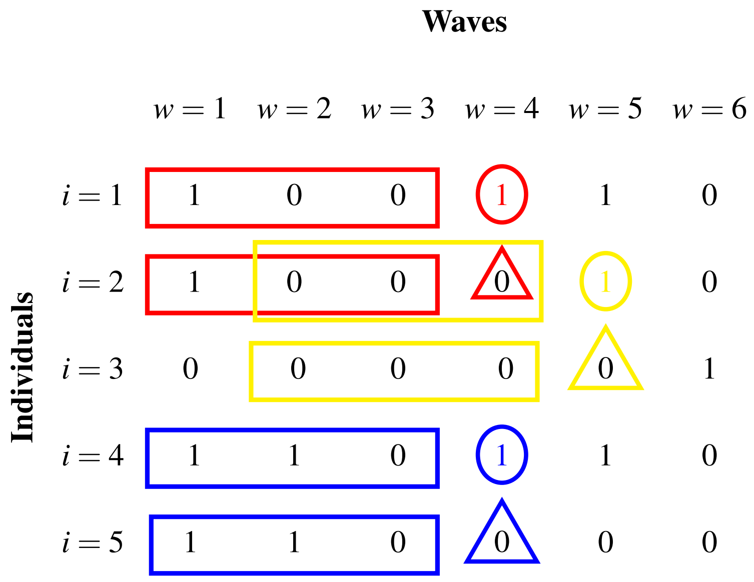

Figure 2: **An example of employment history matching for the case of entry to the labor market** This panel shows how matched sets are made when the number of lags is 3 with one wave lead. Waves are read from left to right. We present the case when the treatment is "entering the labor market", value 1 indicates working and 0 for not-working. Treatment observation (circles) and control observations (triangles) with the same color share the same employment history (rectangles). Likewise, we make separate matching sets for the treatment "exiting the labor market.
Source: Adapted from Imai et al. [10], Figure 2.

Kim JH, *et al. J Epidemiol Community Health* 2023; 77:663–669. doi: 10.1136/jech-2022-220197

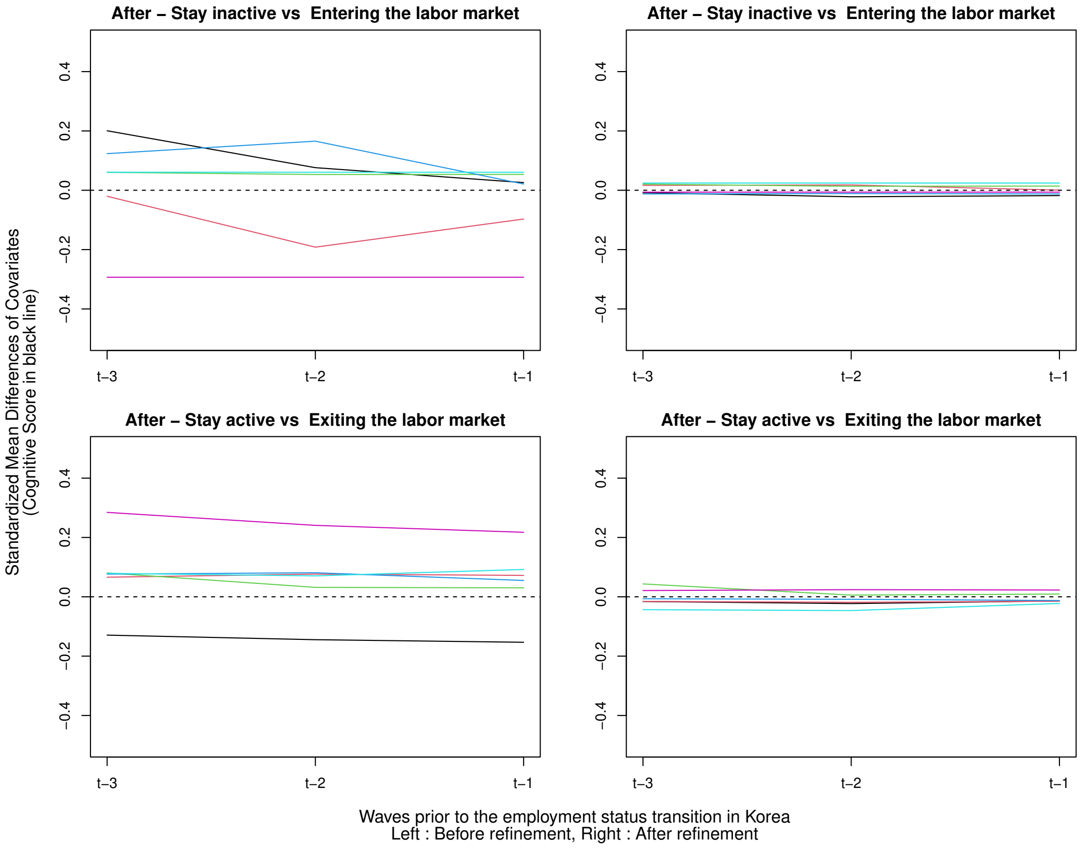

Figure 3: **Covariate balance in KLoSA** Each plot presents the standardized mean difference of covariates over the pre-treatment time period with Korean data. The upper panel represents the balance from entering the labor market and the bottom from the exit. The left column shows the balance before refinement. The right column displays covariate balance after CBPS weighting. The black line represents the balance of the lagged cognitive scores, whereas the colored lines represent highlighted covariates; age (purple), health (red), education (green), asset (blue), and female (light blue).

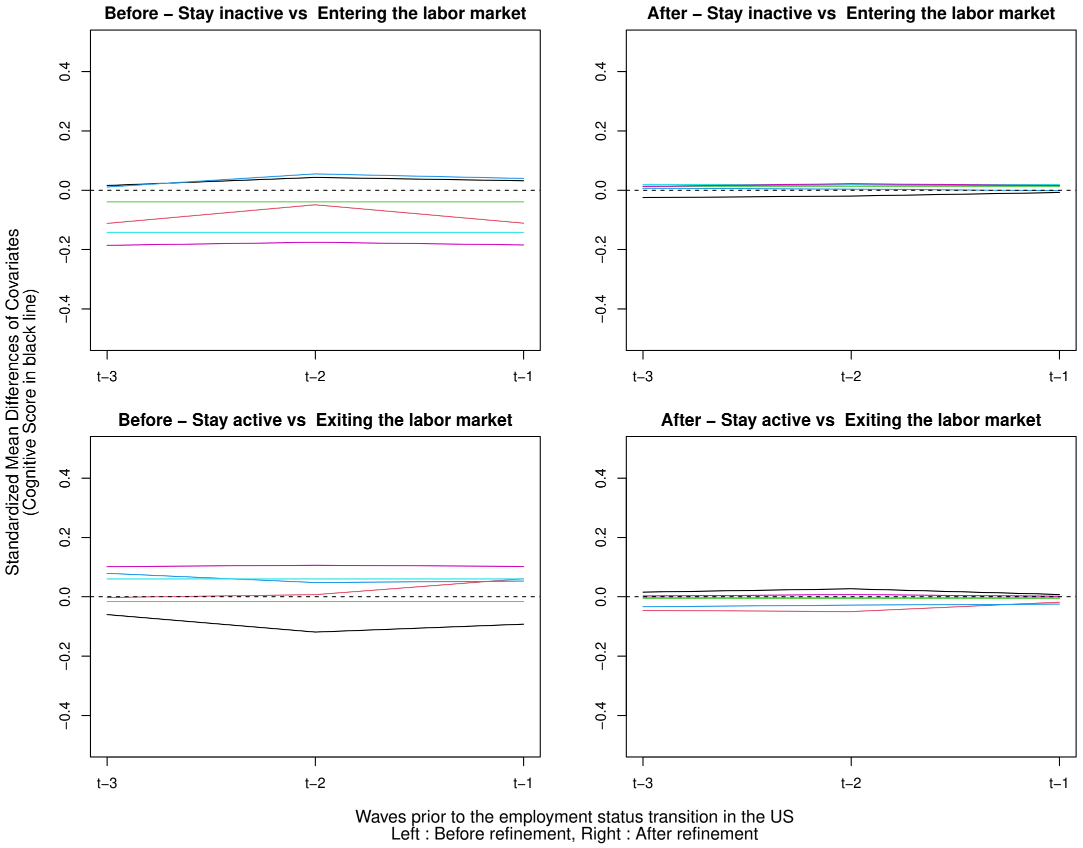

Figure 4: **Covariate balances in HRS** Each plot presents the standardized mean difference of covariates over the pre-treatment time period with US data. The upper panel represents the balance from entering the labor market and the bottom from the exit. The left column shows the balance before refinement. The right column displays covariate balance after CBPS weighting. The black line represents the balance of the lagged cognitive scores, whereas the colored lines represent highlighted covariates; age (purple), health (red), education (green), asset (blue), and female (light blue).

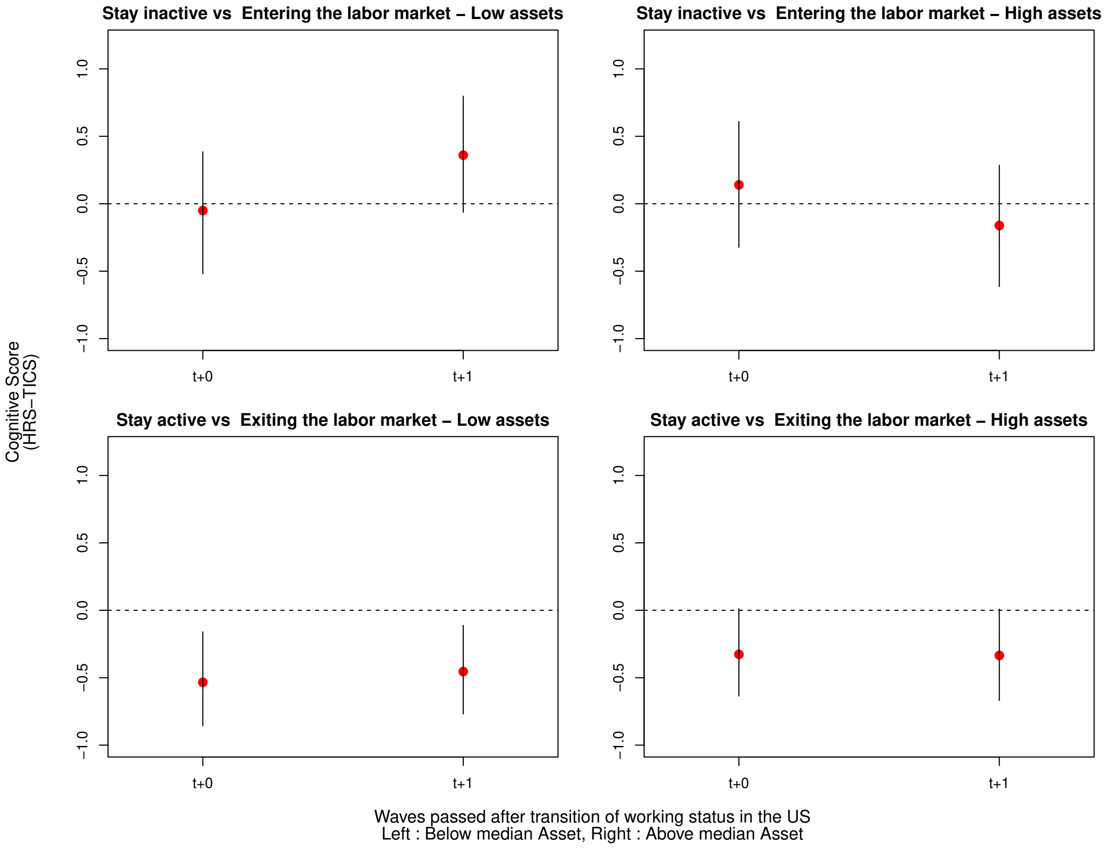

Figure 5: **Subgroup analyses by baseline median asset level with HRS sample.** The estimation results are obtained after matching according to treatment history and CBPS weighting with covariate histories during the three waves before the treatment. The left panel indicates the results from individuals with baseline asset levels below the median, and the right panel above the median. The estimates for the average effects of entering the labor market (upper panel) and exiting (bottom panel) are shown for the period of immediate and one wave after the transition, with 95% asymptotic confidence intervals as vertical bars. CBPS weighting is chosen for its best performance in adjustment.

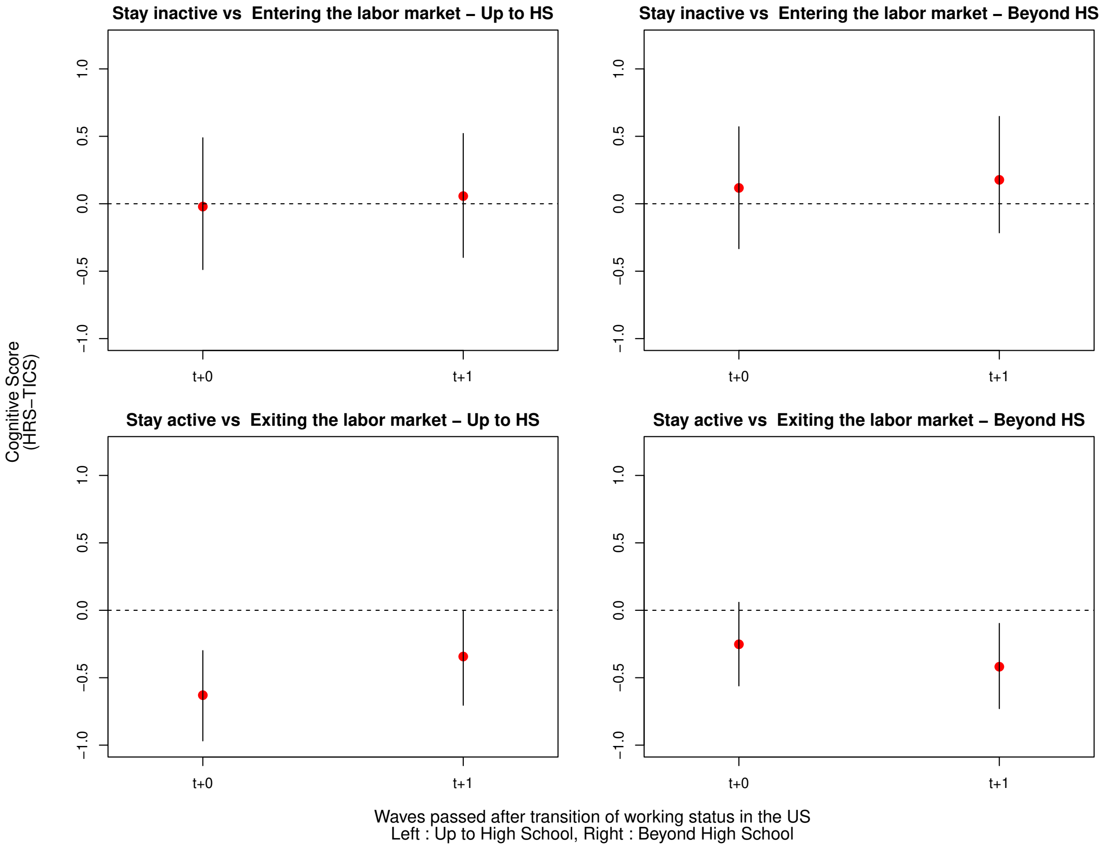

Figure 6: **Subgroup analyses by education level with HRS sample.** The estimation results are obtained after matching according to treatment history and CBPS weighting with covariate histories during the three waves before the treatment. The left panel indicates the results from individuals up to high school education, and the right panel for those beyond high school. The estimates for the average effects of entering the labor market (upper panel) and exiting (bottom panel) are shown for the period of immediate and one wave after the transition, with 95% asymptotic confidence intervals as vertical bars. CBPS weighting is chosen for its best performance in adjustment.

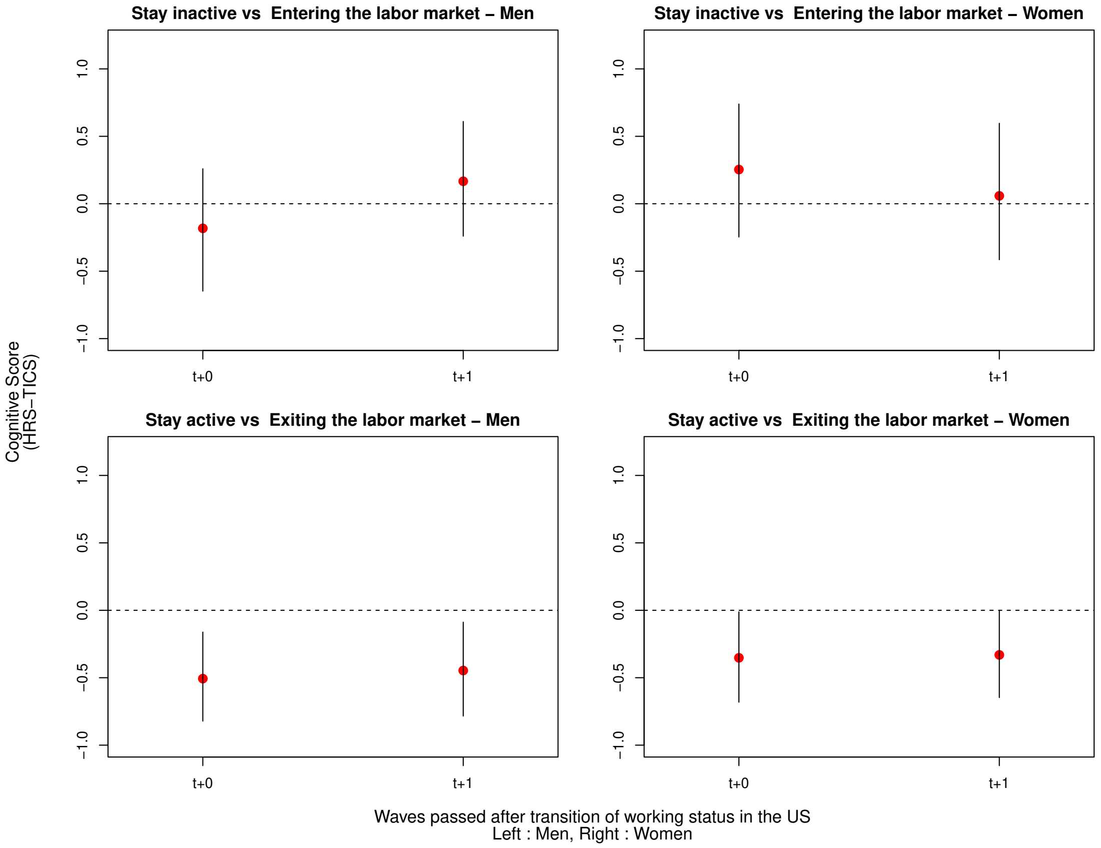

Figure 7: **Subgroup analyses by sex/gender level with HRS sample.** The estimation results are obtained after matching according to treatment history and CBPS weighting with covariate histories during the three waves before the treatment. The left panel indicates the results from the men's sample and and the right panel for the women. The estimates for the average effects of entering the labor market (upper panel) and exiting (bottom panel) are shown for the period of immediate and one wave after the transition, with 95% asymptotic confidence intervals as vertical bars. CBPS weighting is chosen for its best performance in adjustment.

References

[1] OECD. *Pensions at a Glance 2021: OECD and G20 Indicators*. Place: Paris Publisher: OECD Publishing. 2021, p. 224. DOI: 10.1787/ca401ebd-en.

[2] OECD. *Pensions at a Glance 2007: Public Policies across OECD Countries*. Place: Paris Publisher: OECD Publishing. 2007, p. 204. DOI: 10.1787/pension_glance-2007-en.

[3] OECD. "Labour Market Statistics: Labour force statistics by sex and age: indicators". In: *OECD Employment and Labour Market Statistics* (2022). Accessed: 2022-11-29. DOI: 10.1787/data-00310-en.

[4] Cho J, Lee A, and Woo K. "A Comparative Study on Retirement Process in Korea, Germany, and the United States: Identifying Determinants of Retirement Process". In: *The International Journal of Aging and Human Development* 83.4 (2016), pp. 441–467. DOI: 10.1177/0091415016657556.

[5] Lee Y and W.-J.J Yeung. "The Country That Never Retires: The Gendered Pathways to Retirement in South Korea". In: *The Journals of Gerontology: Series B* 76.3 (2020), pp. 642–655. DOI: 10.1093/geronb/gbaa016.

[6] Schwingel A et al. "Continued work employment and volunteerism and mental well-being of older adults: Singapore longitudinal ageing studies". In: *Age and Ageing* 38.5 (2009), pp. 531–537. DOI: 10.1093/ageing/afp089.

[7] Shiba K et al. "Retirement and mental health: does social participation mitigate the association? A fixed-effects longitudinal analysis". In: *BMC Public Health* 17.1 (2017), p. 526. DOI: 10.1186/s12889-017-4427-0.

[8] Feldman D.C. "The Decision to Retire Early: A Review and Conceptualization". In: *Academy of Management Review* 19.2 (1994), pp. 285–311. DOI: 10.5465/amr.1994.9410210751.

[9] Armstrong-Stassen M, Schlosser F, and Zinni D. "Seeking resources: Predicting retirees' return to their workplace." In: *Journal of Managerial Psychology* 27.6 (2012), pp. 615–635. DOI: 10.1108/02683941211252455.

[10] Imai K, Kim I.S, and Wang E.H. "Matching Methods for Causal Inference with Time-Series Cross-Sectional Data". In: *American Journal of Political Science* (2022). DOI: 10.1111/ajps.12685.
